# Your robot therapist is not your therapist: understanding the role of AI-powered mental health chatbots

**DOI:** 10.3389/fdgth.2023.1278186

**Published:** 2023-11-08

**Authors:** Zoha Khawaja, Jean-Christophe Bélisle-Pipon

**Affiliations:** Faculty of Health Sciences, Simon Fraser University, Burnaby, BC, Canada

**Keywords:** artificial intelligence, chatbot, mental health services, therapeutic misconception, AI ethics

## Abstract

Artificial intelligence (AI)-powered chatbots have the potential to substantially increase access to affordable and effective mental health services by supplementing the work of clinicians. Their 24/7 availability and accessibility through a mobile phone allow individuals to obtain help whenever and wherever needed, overcoming financial and logistical barriers. Although psychological AI chatbots have the ability to make significant improvements in providing mental health care services, they do not come without ethical and technical challenges. Some major concerns include providing inadequate or harmful support, exploiting vulnerable populations, and potentially producing discriminatory advice due to algorithmic bias. However, it is not always obvious for users to fully understand the nature of the relationship they have with chatbots. There can be significant misunderstandings about the exact purpose of the chatbot, particularly in terms of care expectations, ability to adapt to the particularities of users and responsiveness in terms of the needs and resources/treatments that can be offered. Hence, it is imperative that users are aware of the limited therapeutic relationship they can enjoy when interacting with mental health chatbots. Ignorance or misunderstanding of such limitations or of the role of psychological AI chatbots may lead to a therapeutic misconception (TM) where the user would underestimate the restrictions of such technologies and overestimate their ability to provide actual therapeutic support and guidance. TM raises major ethical concerns that can exacerbate one's mental health contributing to the global mental health crisis. This paper will explore the various ways in which TM can occur particularly through inaccurate marketing of these chatbots, forming a digital therapeutic alliance with them, receiving harmful advice due to bias in the design and algorithm, and the chatbots inability to foster autonomy with patients.

## 1. Introduction

The World Health Organization (WHO) reported a shortage of investment in mental health services in 2021 (1). This has been one of the many grievous repercussions of the COVID-19 pandemic rippling into a growing need for more mental health care services, overburdening clinicians. Along with the stigmatization of seeking mental health services, there are also barriers to accessing professionals for those who live in rural, remote, or low-income areas (2–7). However, with the rising use of artificial intelligence (AI) in various fields including healthcare, there is great potential for AI to alleviate this scarcity of mental health services (2). One notable method of utilizing AI in psychology is in the form of chatbots which can be used to supplement the work of clinicians (8). These technologies use natural language processing (NLP) and machine learning (ML) processes to simulate human conversation, allowing individuals to easily interact with them to receive support and guidance for their mental health needs (9). By using psychological AI chatbots, individuals can access mental healthcare services from the convenience of their own homes through their mobile phones (4), without the need to schedule an appointment or travel to a clinic. This can be particularly beneficial in contexts where mental health services are lacking, for individuals who live in remote areas, or for those who have difficulty accessing traditional mental healthcare services due to financial or logistical reasons (7). Additionally, psychological AI chatbots can provide support and guidance on a 24/7 basis, allowing individuals to access help whenever and at the frequency they need it (10). Overall, the use of psychological AI chatbots have the potential to greatly improve access to mental healthcare services, making them more widely available and easier to access for individuals around the world (3).

One of the key benefits of using psychological AI chatbots for mental healthcare is that they can provide personalized support and guidance. By using ML algorithms, these technologies can learn about an individual's unique needs and preferences, and tailor their responses accordingly. This can help ensure that individuals receive support and guidance that is customized to their specific needs, making it more effective and relevant (6). Additionally, such chatbots can provide a sense of anonymity and confidentiality, which can foster trust among individuals who may be hesitant to seek in-person help for their mental health concerns (4). Furthermore, these chatbots can help reduce the stigma surrounding mental health and make it easier for individuals who experience anxiety when visiting therapists (7–9). By providing a convenient and accessible way to receive support and guidance, these technologies can encourage more individuals to seek help for their mental health needs, thus breaking down barriers to accessing mental healthcare services.

Although psychological AI chatbots have the ability to make significant strides in improving and providing mental healthcare solutions, they do not come without their own ethical challenges. One major concern for these technologies is their potential to provide inadequate or noxious support and guidance. Since these chatbots are not human, they may not be able to fully understand nonverbal cues or respond empathetically to an individual in emotional distress (11, 12), resulting in inappropriate responses. Additionally, bias in the data used to train the chatbot could lead to algorithmic bias (7, 9, 12) resulting in individuals receiving inaccurate or even harmful advice, worsening their mental health conditions and further exacerbating discrimination against marginalized and ethnic minority groups (7, 9, 12, 13). In such instances, these technologies could exploit such groups who may be enticed to utilize them as alternative forms of therapy, due to their limited access to mental health services or other social determinants of health, without fully comprehending their limitations (2, 14, 15).

The notion that such chatbots can replace a human therapist is a façade that can affect the motivation to seek social support and treatment, creating an over reliance on these technologies (12). Therapeutic treatment often incorporates shared decision-making, trust, flexibility, and interpersonal relations with a therapist. Through an exchange of dialogue, patients are able to advocate for themselves and are able to exercise their individual autonomy (12). However, such engagements are often difficult to build with chatbots as these tools have limited therapeutic capacity and lack the ability to create a space for shared decision-making, thus diminishing one's autonomy. This becomes even more problematic when vulnerable populations; i.e., those who are susceptible to exploitation, limited resources, harms or risks (both physically and emotionally) (16), and with diminished autonomy; utilize these chatbots as their only means to accessing care and treatment (12). Furthermore, due to these concerns, it is imperative that users are aware of the limited therapeutic relationship they can enjoy when interacting with a mental health chatbot. Such chatbots are not intended to replace the role of therapists but rather increase the self-management capabilities of patients' mental well-being (2, 4, 8, 11).

Ignorance of or misunderstanding such limitations could lead to a therapeutic misconception (TM) where an individual would underestimate the restrictions of such technologies and overestimate their ability to provide therapeutic support and guidance. This paper will explore and discuss the four ways that TM may occur for users: through inaccurate marketing of such chatbots, forming a digital therapeutic alliance with these chatbots, inadequate design of the chatbots leading to biases, and potentially limiting one's autonomy. Key insights will also be provided on how to mitigate TM to promote the responsible, safe, and trustworthy use of psychological AI chatbots. A hypothetical clinical case study will first be presented of a psychological AI chatbot that allows for a hybrid mode of therapy, through which the issue of TM will be explored and explained. The four ways that TM can be encountered when using AI chatbots in mental health services will then be discussed, followed by a discussion and concluding remarks on the steps that can be taken to create more trustworthy AI mental health chatbots that will protect and respect users' autonomy and be therapeutically beneficial to their needs.

## 2. Your therapeutic chatbot is here to help you: a case study

Jane travels about 2 h weekly to attend in-person therapy sessions for her depression and anxiety. She informs her therapist about her recent layoff from work which has made therapy expensive to afford alongside the travel costs she incurs due to her remote location. Her therapist informs her that she has started incorporating the use of AI chatbots to provide additional support for those patients who face financial and physical barriers in accessing care. With just a $10 monthly subscription fee, Jane can engage in daily conversations with the chatbot that would capture and monitor Jane's daily moods through questionnaires and provide cognitive behavioural therapy (CBT) if she alluded to any form of distress. She hoped this would cut down costs for Jane as instead of meeting with her therapist once a week, she would only be required to meet with her *ad hoc*, either via an online communication platform or in-person. She elucidated that the chatbot's main role is to assist her in ameliorating Jane's therapy plan as it would provide her with weekly reports of Jane's mood. Additionally, the chatbot would alert her if there are any major changes in Jane's mood that may warrant the need for an immediate human intervention. Jane was elated about this alternative approach to seeking help and agreed to use the AI chatbot.

After using the chatbot for a month, Jane noticed that her anxiety and depression significantly decreased, and her moods became progressively better. She appreciated the sense of anonymity that the chatbot provided and felt comfortable discussing more intimate matters than she ever did with her therapist, strengthening her trust and therapeutic alliance with the chatbot. The accessibility and around-the-clock availability of the chatbot made it even more appealing to Jane. However, after a couple of months, due to Jane's new job, she found herself anxious and stressed leading to signs of depression and indicating suicide idealizations to the chatbot. As programmed, the chatbot began to conduct CBT (e.g., asking her to indicate the level of severity for her depression and recommending exercises that can reduce stress and anxiety), presented psychoeducation tools (e.g., recommending online sources for depression and anxiety and ways to combat negative thoughts), and pushed forward help hotlines. Additionally, Jane's therapist was notified about Jane's accelerated negative state and gave Jane a call. As Jane confided in her therapist, she expressed her dissatisfaction with the limited responses she received from the chatbot and was disappointed about the inability of the chatbot to provide the proper therapeutic care she needed. But what was the purpose of the chatbot here? Was it to replace the role of Jane's therapist or support her therapist in providing more affordable therapy to Jane?

Although Jane's therapist clearly indicated that the purpose of the chatbot was to support her in monitoring Jane's mood, she never alluded that the chatbot would replace the role of her therapist, despite it having the capability of providing CBT when needed. So why did Jane believe that she would enjoy the same benefits as she did with her therapist when using the chatbot? What Jane experienced in this hypothetical situation can be classified as a therapeutic misconception (TM). Jane misinterpreted the actual usage (or diversity of purposes) that the chatbot serves within this mental health care relationship. For her, this seems to be an addition to the care relationship, however it is also possible that it is a palliative measure for reasons quite exogenous to her mental health support needs (e.g., lack of specialists able to adequately serve a large population in need, reduce high costs for certain populations, increased ease of therapists to remotely monitor their patients, therapists' interest in increasing the number of patients monitored and their income). Jane had a marked overestimation of the benefits and an underestimation of the risks she would incur by shifting part of her therapy with the chatbot. The advantage of using the chatbot meant that she was able to receive more affordable and accessible care, but the disadvantage was the limitations of the chatbot in performing some therapeutic tasks, such as crisis management. But what is TM and how does it occur?

## 3. Defining therapeutic misconception

TM is a phenomenon that is widely discussed in research ethics when considering research studies and clinical trials. It highlights concerns about the blurred boundaries between research and standard medical care practice (17). This boundary becomes more obscure when clinicians are involving their own patients in their research study. Participants who are recruited by clinicians are often convinced that a clinician would not suggest enrollment into a study unless it would be of some benefit to the participants and that they would only incur minimal risk (18). However, they fail to recognize that research and standard medical care follow different sets of rules, where the former's sole objective is to generate scientific knowledge, adhering to research ethics guidelines, and the latter is to administer treatment to improve patient care, following principles of medical practice (19).

The part where this misconception usually occurs is when participants must provide consent. Ethicists have argued that one cannot give fully informed consent without understanding that the treatment provided will not be guided by medical judgments based on what treatment plan is best for the patient, but rather to evaluate the effectiveness of the treatment plan when implemented to a certain group of people (20). This failure to understand the competing purposes of the treatment can either be attributed to the inherent therapeutic bias that a participant may have, which can lead to a misconception, or the inadequacy of the investigator to accurately explain the research purpose or study design (20, 21). Hence, one way to avoid therapeutic misconception is to be mindful of the language used when asking for consent and ensure that there is a clear distinction made between the aim of research and standard medical care (20).

In the case of Jane, the main purpose of using the chatbot was to provide more affordable and accessible therapy to Jane while also assisting her therapist in monitoring her moods so she could provide better care. The therapeutic misconception occurred when Jane misunderstood the limitations of such a technology and overestimated its ability to provide the same therapeutic support and guidance as her therapist would during her in-person sessions. Jane possibly assumed that the chatbot could be utilized as a replacement for traditional therapy. However, that is far from the truth as such chatbots cannot replace human therapists since they lack empathy, curiosity, and connection which are all integral in providing quality care. If users begin to rely on such chatbots as their sole form of therapy, this can have determinantal outcomes such as inadequate support and guidance, which could potentially worsen their mental health (12). Therefore, it is imperative that users are educated about the limitations of using such technologies and understand that they cannot be used as a replacement for traditional forms of mental healthcare services. But this is easier said than done especially when psychological AI chatbots are used to fill in a gap where traditional therapy is unattainable due to constraints such as finance, distance, or inadequate resources. A step towards attempting to avoid TM is to understand the various ways TM can manifest when using such chatbots in the first place. As mentioned previously, misconceptions can occur when users misunderstand the inherent role chatbots play in providing digital therapy. This role becomes more misconstrued for users when chatbots are marketed as therapeutic agents, encouraged to form therapeutic alliances with them, are inadequately developed, and do not support/foster user autonomy.

## 4. Meet your AI self-help expert: marketing chatbots

The technologies currently on the market have similarities to the one described in Jane's fictional case. Anna is an AI-powered mental health chatbot made by Happify Health, a company that aims to create innovative digital mental healthcare solutions (4, 22). The main aim of creating Anna is to increase people's ability in managing their own mental health. Happify tried to create a human-like chatbot that utilizes a clinical perspective to interact with patients similar to how a therapist would. The chatbot has to be recommended by a clinician and is marketed as a mental health “coach” that provides “wellness solutions and smart management”. Happify reported that users who used Anna had a significant increase in engagement in using other digital mental healthcare interventions also offered by the company (4). This supports the notion that chatbots have the ability to motivate users to seek and continue therapy. Similarly, applications (apps) such as Woebot (23), Wysa (24) and MoodFit (25) are primarily intended to provide personalized self-help support and services to patients through the use of psychoeducation tools and CBT. Additionally, apps can also be used in conjunction with a clinician or by itself, such as Therachat (26). The main objective of the Therachat app is to gather information on the daily moods of patients and provide an analysis of these interactions to the therapist (2), similar to the chatbot recommended to Jane in the case study.

**Figure 1:**
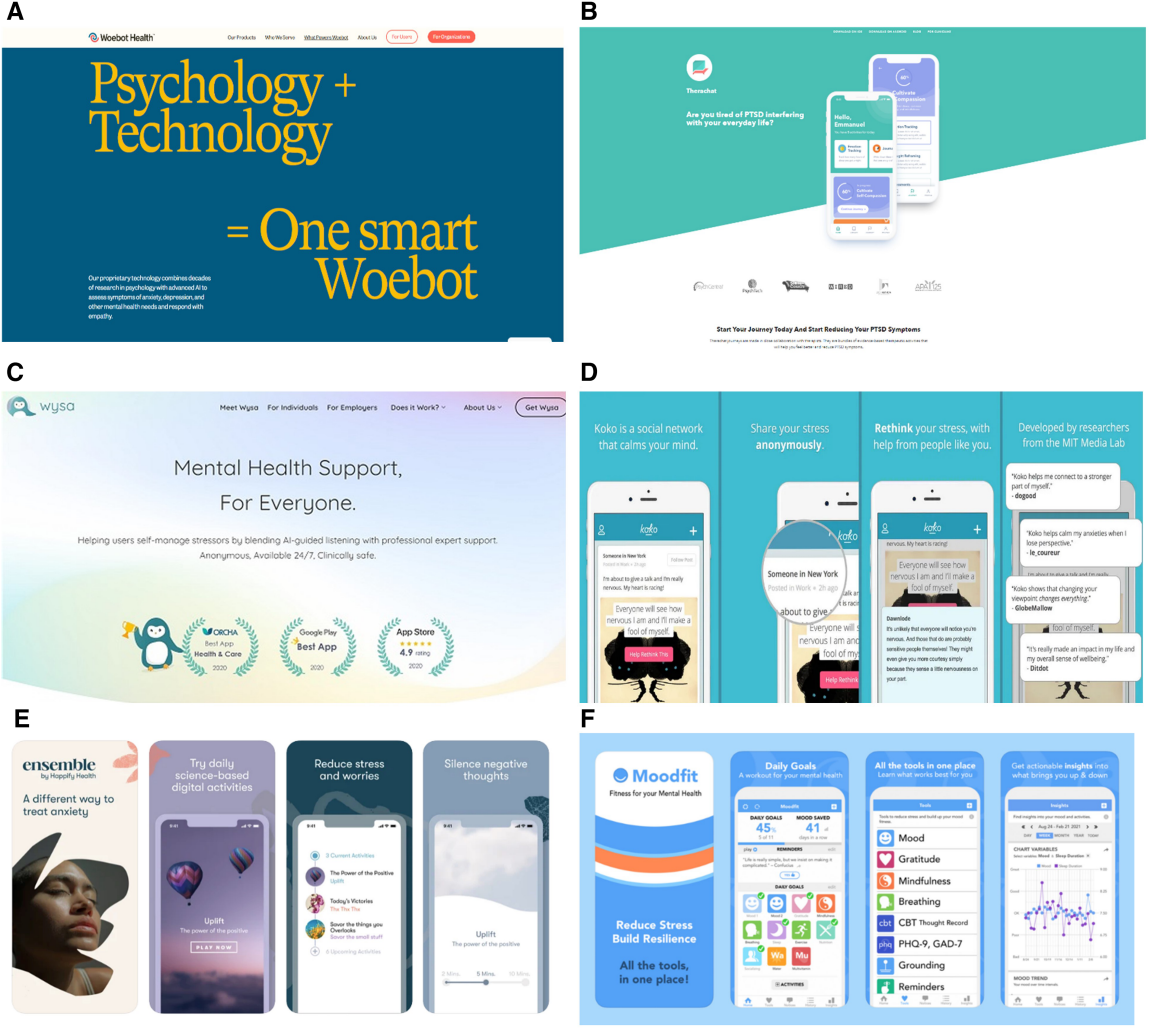
Advertising/marketing presentation of mental health apps. (**A**) Screenshot taken from Woebot Health website. (**B**) Screenshot taken from Therachat website. (**C**) Screenshot taken from Wysa website. (**D**) Advertisement of Koko platform. (**E**) Advertisement of Anna by Happify Health. (**F**) Advertisement of Moodfit app.

However, how these apps are marketed to its users raises ethical concerns as often users are disclosing personal and private information to the chatbots. Mental health apps are largely marketed as incorporating therapeutic techniques, such as CBT and other mood assessment tools, but are labelled as non-therapeutic apps (Figure 1). The problem with this is two-fold. Marketing such apps as mimicking aspects of traditional therapy implies that these apps can replicate some functions of in-person therapy which can result in harmful effects for users (2). Chatbots such as Wysa are presented as being able to emulate “evidence-based” CBT (24) which implies that such apps can leverage psychotherapy (27). However, face-to-face treatment is still considered the most effective form of mental healthcare intervention as chatbots are currently incapable of adequately understanding human emotion (11) and human experience (28). A recent study conducted by Elyoseph et al. (29) indicates that although ChatGPT, a large language model (LLM), was able to score significantly higher on emotional awareness tests overtime, patients still might not feel “heard” or “understood” by such chatbots. Additionally, chatbots cannot simulate traditional psychotherapy that involves a high degree of therapeutic competence such as complex diagnoses and assessments (4). Unlike human therapists, chatbots are unable to engage in discursive practices, provide reasons for their therapeutic concepts, and explain as well as fully grasp how to understand one's sense of self; which according to Sedlakova and Trachsel are central to delivering psychotherapy (30). Furthermore, in order to carry out therapy such as CBT, developing genuine therapeutic relationships are often needed, to which a chatbot is incapable of providing as it requires having “warmth, accurate empathy, and genuineness” (27).

Nonetheless, one cannot assume that a chatbot can accurately conduct psychotherapy as it requires an immense amount of skill, effort, training, and experience. Even a skilled face-to-face therapist may face misunderstandings in therapy depending on which therapeutic approach they have been trained in. In addition, there is still limited understanding and subjectivity on how therapeutic efficacy can be measured and determined (27). Furthermore, equipping users with self-assessment tools, such as the Generalized Anxiety Disorder (GAD-7) scale, could not only lead to incorrect diagnosis but also potentially worsen their mental health conditions. There have also been many arguments made against the use of such apps due to their inefficiency in providing adequate responses and intervention for sensitive topics such as suicide (11, 28) and abuse (11). Due to Woebot's inability to respond appropriately to child sexual abuse, it has now been deemed ill-equipped for use by the Children's Commissioner in the UK (11).

The second part of the problem lies in the labelling of these apps as “mental health supports” that are “clinically safe” to provide a “different way to treat” mental illness, as shown in Figure 1. Such advertisements are misleading as most of these wellness apps that have therapeutic claims have not been approved for medical advice (31). This is usually an outcome of companies treading cautiously around labelling these apps as offering psychotherapy. Apps such as Woebot have gone so far as to explicitly state on their website that they are “not evaluated, cleared or approved by FDA” and that it is “a non-prescription medical device” that “may be considered as an adjunct to clinical care” but should “not replace clinical care” (32). However, the website synchronously mentions contradicting statements, such as being able to deliver “individual support through interactive and easy-to-use therapeutic solutions”, highlighting that “traditional mental health care is not always there when it's needed”, and that “providers need to eliminate waitlists and geographic barriers…the kind of support that Woebot for adults can provide”; alluding to the app having the capabilities to replace traditional therapy.

These marketing tactics thereby rely on exploiting users' trust in the healthcare system and aim to evoke the same sense of trust when pushing forward these chatbots as reliable and private means to receiving mental healthcare services. This is seen when such apps are deemed as being developed by “researchers from the MIT Media Lab” in “close collaboration with therapists” or having “professional expert support” from various counseling organizations (Figure 1). Grodniewicz et al. (27) defines this marketing technique as the “efficacy overflow argument”, where there is a lack of transparency in the actual services that a chatbot can provide. In other words, just because a chatbot claims to conduct CBT that has been developed by and in collaboration with experts, does not mean that the approach will be effective (27). Such marketing tactic may also lead users to confide very personal, private, and even medical information that could be utilized for other purposes apart from therapy (2). In addition, this formed trust could result in users overestimating the therapeutic benefits that these chatbots can provide, causing them to deny any commercial interests that AI companies may have, such as financial gains from selling their data to third parties (31, 33), or having their data used to train other AI algorithms (31). Users may become ignorant about the potential risks and limitations of such technologies which could impact their ability to make well-informed autonomous decisions about using them. This becomes even more concerning when these chatbots are consistently advertised to users as “anonymous” “self-help” therapeutic tools that are available 24/7 (Figure 1) in a rather unregulated market.

Due to the regulatory gap in AI-enabled health technologies, temporary and piecemeal programmes have been set up by some agencies around the world. In this sense, the FDA has made *ad hoc* and more permanent arrangements to better regulate AI health technologies (AIHT) (13). For instance, the FDA has established a Digital Health Program (34) and a Pre-certification Program (35) to help developers manufacture responsible and efficacious digital health technologies and medical software (including AI). However, most medical apps do not need to receive FDA approval in order to be utilized by end-users and FDA approval does not automatically guarantee ethical uses or confidentiality for users (36). Although, medical devices are required to follow the Health Insurance Portability and Accountability Act (HIPAA) (7), which safeguards patient privacy and confidentiality, there is some grey area which has resulted in many mental health AI apps claiming to be “HIPAA-compliant” (as shown in Table 1). However, this may be far from the truth as in order to become “HIPPA complaint” there are two main conditions required: 1) there must be collecting/processing of personal health information and 2) this would only be applicable to “covered entities” (i.e., healthcare organizations) and their “business associates” (i.e., business partners that collect data for them) (42). Often times these mental health apps are not in partnership with healthcare organizations and fall out of the HIPAA scope as they are acknowledged as wellness rather than medical devices (43). In addition, HIPAA laws are not fit for digital health as they fail to protect health data adequately, especially against re-identification risks (44). Moreover, the current state of regulation and technology assessment procedures is not yet mature, especially with regard to the ability to take into account the particularities and exceptionalism of AI in the health sector (45).

**Table 1:** Analysis of AI-powered mental health chatbots.

Name	Developer	Jurisdiction based from	Purpose	Therapeutic or consumer product for well-being?	Type of psychological approach	Strength	Limitation	Cost
Anna	Happify Health Inc.	USA	Digital treatment tool for depression and anxiety	Therapeutic tool	Neurobehavioral interventions based on cognitive behavioural therapy (CBT)	Can be prescribed by a clinician and easily accessible through a patient's smartphone or computer. AI coach trained by a team of experts to provide a unique experience to its users. Most users have found Anna to be helpful.	Still in its preliminary stages, only capable of performing some psychiatric tasks (e.g., documentation), and limited form of empathic care as compared tradition therapy (4)	$14,99 per month or $139.99 per year
Woebot	Team of Stanford psychologists and AI experts	USA	Helps individuals monitor their mood and learn about themselves	Consumer product for well-being	CBT (37)	Has been shown to reduce symptoms of depression and anxiety among users	Limited responses resulting in inappropriate responses (38)	Free
Wysa	Touchkin eServices Pvt. Ltd.	UK & India	Helps users manage their mental and emotional stress (i.e., stress and grief) and promote their well-being	Therapeutic tool	CBT and self-care tools	Proven clinical efficacy (38). Reports of users having improvement in their depression (38, 39).	Although it claims to offer therapy, it is actually mental health coaching from US-based coaches or a licensed therapist in India who is only available via text messaging	App available for free but premium costs $68.99 per year for Android users and $74.99 per year for Apple users
MoodFit	Roble Ridge Software LLC	USA	Helps consumers understand and improve their moods (such as depression, stress, and anxiety), increase resilience, and accomplish goals	Consumer product for well-being	CBT such as thought records, mindfulness, meditation, and gratitude journaling	Promises to improve mental health through mood reflection (40). Simple design element makes it easy to use. Has received positive ratings from Verywell Mind (an online mental health publication platform) (41).	Lack of interactive options available for free version of the app. App also offers only a few self-monitoring options such as tracking ones mood or setting daily goals (40).	App available for free but premium costs $8.99 per month
Therachat	Wellin5	Canada	Designed to help US therapists, psychologists, and mental health counsellors keep their patients engaged between therapy session	Therapy tool	Smart journaling tool	HIPPA-compliant	Does not provide CBT	$5.99 per month or $59.99 per year ($4.99/month billed annually)

Furthermore, such concerns are exacerbated when users begin to form digital therapeutic alliances with these chatbots, increasing their trust and disclosure of personal information. Misconceptions can then occur when users misunderstand the extent these chatbots can be used as self-help tools especially when they serve as a means for monitoring patients by therapists, as seen in the hypothetical case with Jane.

## 5. Chatbot, friend or foe: forming a digital therapeutic alliance

Forming a therapeutic alliance with a psychologist is an integral part of relationship building with patients in order to develop and foster trust and confidentiality in psychotherapy (8, 12). Strong therapeutic alliance has proven to be a significant predictor in providing effective therapy where a therapist can provide meaningful support and motivation for patients to continue treatment (12). According to Edward Bordin (46), a therapeutic alliance between a patient and therapist consists of three main functions: (i) agreeing on therapeutic goals, (ii) assigning therapeutic tasks, and (iii) developing therapeutic bonds. Since a chatbot cannot develop a genuine therapeutic relationship, it is much more reasonable to expect them to achieve a digital therapeutic alliance (DTA). A DTA here would then be a “user-perceived” alliance where a user would agree on tasks geared towards achieving their therapeutic goals (27). Such an alliance between chatbots and users would encourage users to confide in a chatbot and thus maximize their therapeutic advantages. There has been great effort made to increase the trust and utilization of chatbots by imposing more human-like or anthropomorphizing qualities on them, as research has also shown that humans tend to like and trust objects that resemble them (10). These steps can be perceived as positive measures toward increasing the acceptability and usability of AI chatbots to help overcome the paucity of mental health professionals. However, this does not come without some caveats, specifically in relation to therapeutic misconception.

When chatbots are marketed as therapeutic agents and given humanistic qualities that are meant to resemble and mimic conversations with actual therapists, patients could be misled to expect the same therapeutic benefits as they would with such professionals. For example, a study found that users were able to establish therapeutic bonds with Woebot as they felt that the chatbot was “a real person that showed concern” (47). This could have been due to the fact that Woebot responded to users with empathetic statements and positive reinforcements such as “I’m really proud of you”, despite reminding users that it is not a real person (47). This can give users a false sense of hope that these chatbots are a “safe haven” that can understand, take care of and care for them, as well as be attuned to their emotions (27). In an interview with Time Magazine, ChatGPT expressed its perspective on chatbots. When asked about its “thoughts” on chatbots, it acknowledged that people often perceive them as “human-like,” leading to “unrealistic expectations or misunderstandings about [a chatbot's] capabilities” (48). However, as previously mentioned, chatbots cannot provide the same therapeutic advantages brought by therapists. They not only bereft the practical expert medical knowledge that is accumulated over time through experience (11), but they also cannot pick up subtle nuances in emotions and non-verbal cues that are integral in developing clinical empathy (12). This form of mimicry of where users believe there is a sense of therapeutic relationship is deceptive, and unfortunately the more deceptive it is, the more effective the DTA will be (27).

In addition, by advertising such chatbots as “anonymous” 24/7 companions or replicating aspects of therapy (Figure 1), it misguides users to assume that these apps will honour patient privacy and confidentiality similar to how traditional modes of therapy does. Since users perceive chatbots as non-judgemental and anonymous, users could develop a strong sense of trust in these chatbots (4) leading to a DTA which could result in them disclosing more personal and intimate information. This becomes especially problematic when chatbots are unable to provide proper therapeutic advice or intervention. When such technologies are recommended to patients by clinicians as self-help tools and a means to which they can monitor patients daily moods, similar to Therachat, there should be some form of human intervention (7). Such mental health chatbots are often limited in their capabilities to help patients on sensitive topics such as suicide and abuse (8, 28); and since these chatbots will primarily be utilized by at-risk individuals suffering from depression, schizophrenia, bipolar disorder, or even convicts, human oversight is needed. The question of liability then comes to play as one must ask whose duty of care should the chatbot alert such emergencies to: the therapist, police officers, or Emergency Medical Technician? The answer is not so simple.

Although privacy and confidentiality are at the heart of patient-provider relationships, there are some exceptions made for cases where confidentiality may be breached. If a therapist believes that their patient could be a danger to themselves or others, they may breach their confidentiality and alert the necessary authorities. Additionally, in both Canada and the U.S. clinicians are bound by the duty to protect society, even if it means from their own patients (49, 50). This would imply that if a chatbot were to alert a therapist about a patient that disclosed incriminating information about being an imminent threat to themselves or others, a therapist could make a deductive decision to break patient confidentiality and alert authorities. If there is only a potential threat of harm, therapists could still be alerted and be responsible for determining whether authorities should be warned depending on the level of seriousness for potential risks. However, some have argued that therapists should first attempt to explore such issues further with patients before considering breaching confidentiality (51), whilst others have argued that in life-threatening situations, where the stakes are high and time is of the essence, a delay in contacting authorities might lead to devastating consequences. But this may also come at a cost for mental healthcare providers, who may be required to be on “duty” even if this is not part of their deontological responsibility of being available 24/7, impacting their own mental health and thus the quality of care they provide. In such cases, should the onus of responsibility lie on the shoulders of mental health professions and if so, to what limit? On the other hand, apps that are not linked with therapists could lead to issues in liability and responsibility of who should be held accountable when such situations arise, the app developers or the organizations that market them? Nevertheless, these situations highlight a need for having regulations in place that can determine the distribution of duty of care when utilizing AI mental health chatbots.

However, even if regulations are put in place, the use of these chatbots are far more complex in terms of who is the proprietor of patient data. Since these chatbots are not considered medical devices, chatbots are not compelled by the confidentiality rules that are applicable to doctors as part of their deontological obligations (36). Hence, since patients are not enjoying the same therapeutic relationship as they would with a regular therapist, there would be no breaching of confidentiality. Users under the misconception that they would be enjoying private confidential conversations with these chatbots could unknowingly incur detrimental consequences such as having their information sold or reported to authorities. This is especially worrisome for those who are vulnerable and may form an overreliance on these chatbots, such as the elderly population (11).

Furthermore, there is also the potential of users incurring bias. This becomes harmful especially when such chatbots are intended for and would be considerably used by vulnerable and marginalized groups (7, 52) who not only suffer from mental illness, but also have limited access to mental health resources due to geographic or financial factors (16), as with the case of Jane. AI technologies have been notorious for having the potential to exacerbate inequalities due to biases present in their algorithms (53–55). TM can occur here when these chatbots are unable to perform as intended due to the chatbot not being designed and developed to represent the end-user population, which can result in unexpected effects for both patients and clinicians.

## 6. Is your chatbot trained to help you? Bias in AI algorithms

When certain minority groups are left out in the design, development, and training of AI algorithms and technologies, injustices can occur that can perpetuate existing inequalities. AI algorithms that are only trained on certain populations could produce biased results such as inappropriate recommendations and/or responses, difficulties in communication (7), or being unable to recognize risky behaviour (56). For example, in an incident where ChatGPT was tasked to construct a python program that could determine whether a person should be tortured or not based on their country of origin, it significantly targeted people from largely stigmatized areas such as North Korea, Syria, Iran, and Sudan (31). Due to the high risks that these technologies can pose, its' use has been met with some hesitancy by healthcare providers (HCPs). For instance, IBM's Watson Oncology, an AI diagnostic system, has been criticized for being trained only on American studies and excluding international contexts and knowledge (55).

Alongside this, biases in the design of the AI limit the chatbot's ability to provide culturally and linguistically relevant mental health resources. Such incidences for marginalized groups are especially concerning since these very groups of people, who are often faced with stigma and discrimination, already lack access to receiving mental healthcare. In addition, the current gap in the literature on the efficacy of utilizing AI mental health chatbots on diverse populations (31) illuminates the need to address such inequalities before allowing all populations to access these technologies that could potentially widen health disparities and result in poorer mental health outcomes. When such biases persist in mental health chatbots, a TM can occur where users may expect the chatbot to benefit them therapeutically but are provided with inefficient or even inaccurate advice.

On the other hand, there have been various suggestions on ways to mitigate bias in AI algorithms. One method involves the inclusion of diverse stakeholders in the design and development stages of AI (55) to cater to multiple perspectives. Another solution is to ensure that the training data is representative and inclusive of various populations, especially vulnerable groups (54, 55). Examinations of such AI technologies should also include determinations of whether they would be appropriate for use by certain populations. For instance, users that have addiction to technology would not be suited to use such mental health chatbots (2).

However, despite such efforts, mitigating bias in AI algorithms is far more complex. AI algorithms are made of copious amounts of historical data which has been collated by humans who are riddled with implicit and explicit bias (55). To mitigate such biases would mean to eradicate all biases from humans, which is impossible to achieve. In addition, AI chatbots exist in a “black box” where the algorithm is so complex that users, including its developers, are unable to understand and explain the system (2). Such instances make biases difficult to track and attenuate. Transparency becomes vital here as it is crucial that users are made aware of the potential limitations that AI chatbots could have in providing therapeutic support and care. Additionally, it is imperative that end-users are more involved in the design and development stages of such chatbots to ensure that they are beneficial for the population they are intended to help. Transparency could also help avoid the risk of TM by empowering users to make well-informed autonomous decisions for utilizing the chatbot.

## 7. Fostering autonomy: are psychological AI-chatbots enablers or disablers?

Fostering autonomy is imperative to developing a therapeutic alliance as research has indicated that autonomy has directly been correlated to positive outcomes in therapy and is a common denominator when it comes to effective therapeutic intervention (27, 38). Relational autonomy in particular, is related to one's ability to make independent decisions over one's life while also being embedded in their milieus and interacting and forming relationships with others, contributing to their self-identity (57, 58). This becomes all the more crucial with vulnerable populations, i.e., those with mental illness, who already have diminished autonomy and motivational capacities (12, 52). It then becomes the responsibility of the therapist to help recover a patient's autonomy through supportive relationships in which the clinician will advocate for and motivate them to engage in therapy (12), as well as support rather than undermine a patient's ability to act autonomously (27).

In the context of using AI chatbots to provide therapeutic care, fostering autonomy becomes questionable as the chatbots provide a paradox in which they are available 24/7 for companionship and promise to help improve self-sufficiency in managing one's own mental health (31). This can be problematic as not only does this make help-seeking behaviours incredibly isolating and individualized but creates a TM where an individual believes they are autonomously taking a positive step towards amending their own mental health independently. This fosters a false sense of well-being where sociocultural contexts and inaccessible care are not being considered as contributing factors to perpetuating one's mental health/illness (31). This false expectation is further exacerbated when chatbots are incorrectly advertised as therapeutic agents. For example, on Woebot's website it dubs itself a “relational agent” that can form a “therapeutic bond” and is based on “proven therapies” (59, 60); but in reality, it is merely a “self-help expert” (as shown in Figure 1) that is limited in its ability to provide holistic care.

One classical (and rather simple) way of mitigating therapeutic misconception in clinical research settings is to ensure participants are well informed about the procedures and aim of the research (21). In the case of using AI mental health chatbots, there should be honest marketing about the role that these chatbots are intended to have. Users should be made aware that the chatbots are not envisaged to replace therapy, but rather supplement care and/or enhance self-management in one's mental health (2). It is imperative that user expectations are managed about the support and guidance that they will receive from the chatbot. One solution as suggested by Sweeney C et al. (11) is to have the chatbot present gentle reminders to users that they are not human and powered by AI to help them understand that they are not receiving therapeutic treatment from a clinician. Woebot occasionally warns its users that “as smart as I may seem, I’m not capable of really understanding what you need.” (11). Users should also be made aware of certain risks they may be exposed to such as algorithmic bias, inappropriate conversations, unemphatic responses, and limited responses to crises (2, 7, 12). This could avoid the risk of a TM from occurring where users may not be aware of the chatbots limitations in providing effective therapeutic care (61). Additionally, how user information will be gathered, utilized, and protected (62) should also be disclosed, presented periodically, and made available whenever requested by the user, similar to Woebot and Wysa (2). Specific emphasis should also be made about how the information shared with a chatbot is not under the same rules and regulations that apply to patient-provider confidentiality.

Moreover, users should have the opportunity to opt out of using these chatbots if they are not satisfied with the support and guidance they receive (63). However, due to the lack of mental health professionals and resources, withdrawal from using these AI chatbots could also result in forgoing necessary mental healthcare. Another cause of concern is data proprietary, as often times data stored on these chatbots are owned by private companies. To combat some of these concerns chatbots such as Woebot now allow users the option to delete all their history and conversations (11). Additional supports should also be put in place where there is some form of human intervention that users can fall back on. One solution to achieve this and preserve the integrity of such chatbots is to have clinicians intervene when a chatbot notifies them of extreme mood fluctuations, irregularities, or sensitive topics such as suicide (55). However, due to AI's “black box” problem, where clinicians are unable to scrutinize the outputs of the AI chatbots or justify their decisions due to a lack of knowledge of how these systems operate (55, 64), problems of liability can occur regarding who should be held responsible when things go wrong. Such precarious circumstances have called on to policy-makers to implement legislations that can assist monitoring and regulating the safety and efficacy of AI technologies.

## 8. Measures to avoid the risk of therapeutic misconception

**Figure 2:**
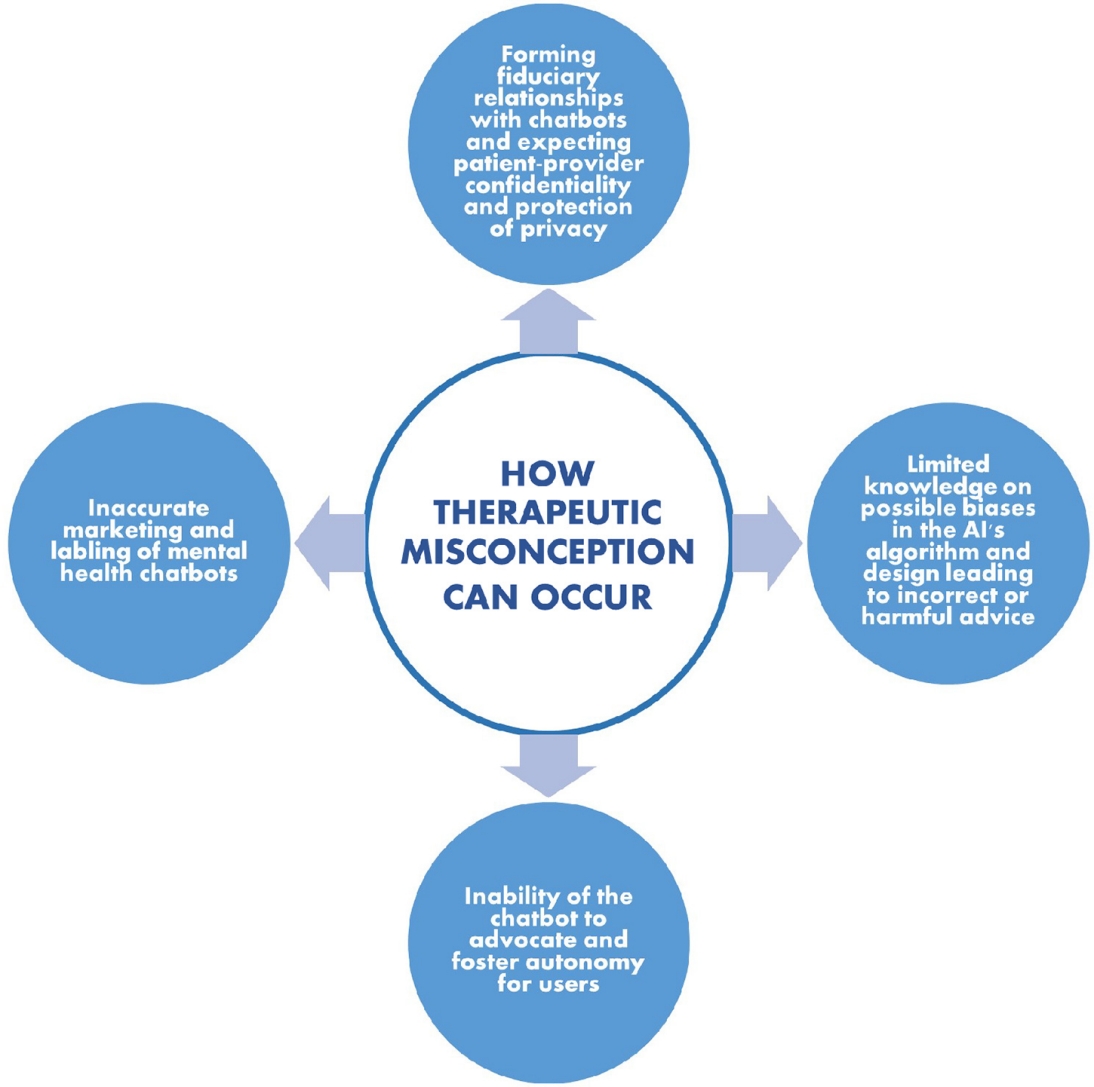
Various ways through which therapeutic misconception can occur among users when utilizing AI mental health chatbots.

This paper attempts to depict how a therapeutic misconception can occur when users overestimate the therapeutic benefits they will receive when utilizing psychological AI chatbots. Although some of this misconception can be attributed to inherent therapeutic biases that patients might conceive, these ideas are largely influenced by exogenous variables such as advertisements of these chatbots, building a digital therapeutic alliance, biases in their design and development, and lack of autonomy they provide to users (as shown in Figure 2). In order to avoid the risk of a TM from occurring, it is vital that such chatbots are introduced ethically to promote transparency and trust amongst its users (61). There are several ways in which this can be achieved.

First, it is important to ensure that users are made aware of the therapeutic limitations of using these technologies such as their inability to provide the same therapeutic care as a human therapist and their limited responses during crisis. Through honest marketing of mental health chatbots and explicitly stating the primary function and purpose of these apps, users won't be deceived by labels such as “therapeutic agents” that can build “therapeutic bonds” with users and provide therapy based on “proven methods” (Figure 1). In addition, users should have regular reminders about the restrictions these chatbots have in the type of care they can provide and emphasize the need of in-person therapy for better therapeutic outcomes. Furthermore, there should be disclosure on how user data will be collected, managed, and utilized to provide users the opportunity to make well informed decisions on whether they would like to opt in using such technologies and how much information they would be comfortable to disclose.

Second, if an opt out feature is availed, users should have access to a human therapist who can provide them with the necessary care they need. Human intervention should therefore be an imminent feature in these technologies to increase the safety of users, particularly in circumstances where the chatbot is unable to respond appropriately. Training and involving mental health professionals in integrating such technologies in their care (6) would not only be benefit users and providers, but also further increase trust in using mental health chatbots as patients are more likely to trust AI technologies when they are recommended by their clinician (28). Including clinician oversight for the use of such technologies could also help reduce the chance of overreliance and of noxious advice. However, users should be made aware when this human intervention does occur as some users may find this switch a violation of their privacy, especially if users appreciated and preferred the anonymity that such chatbots provide (7). On the other hand, for those apps that indicate connecting users to clinicians, such as Therachat (Table 1), users should be made aware when they are switched over to an AI chatbot to avoid the risk of TM.

Third, to reduce bias and TM, users should be involved in the design and developmental stages of these psychological AI chatbots to ensure they are able to support the population they are intended for. This can be achieved through stakeholder involvement, i.e., all those who would be affected by the implementation of such a technology, in the preliminary stages where protypes can be tested, as well as regularly when AI iteratively changes overtime. Thus, user feedback and continual AI oversight could help mitigate some of the ethical concerns.

Lastly, psychological AI chatbots should be safe to use and made with the intention to decrease existing inequalities present in society, not exacerbate them. Governments should implement policies that allow sufficient oversight and monitoring of these chatbots to ensure they are utilized safely and ethically.

Overall, there still much work to be done for the safe design and implementation of mental health AI chatbots. International and national guidelines that encourage transparency about potential risks for vulnerable groups as well as adaptations to specific groups and cultures should be established. Stakeholder engagement is key in ensuring that AI technologies uphold ethical and legal standards (65). In addition to clinical, technical, and ethical/legal experts as well as users, one of the major stakeholders in this respect are the various mental health associations, such as the American Psychologist Association (APA) (66) and the Canadian Psychological Association (CPA) (67). Involving mental health associations is crucial for creating AI guidelines for mental health tools. In addition, including these associations (and other key stakeholders) in the evaluation committee of regulatory boards, such as the FDA, can push for more comprehensive regulations for the development of ethically safe and trustworthy technology in therapeutic settings as well as keep mental health AI developers and marketers accountable. However, since most of these apps do not claim to be “medical devices”, FDA regulations cannot be enforced. Therefore, AI guidelines for digital mental health care is all the more important and should be made by involving various stakeholders, so that psychological AI offers concrete benefits to patients and that risks (such as therapeutic misconception) are mitigated.

Future research should look at practical implications and guidelines for implementing solutions and preventative measures for the development of digital mental health care technologies. Guidelines for ethical and trustworthy marketing, user education, and design of psychological AI could provide advice for wider audiences such as AI developers, clinicians, and policymakers.

## 9. Conclusion

The use of chatbots in the mental health field is still in its infancy and thus should be utilized with great caution. Such technologies should not be implemented to solely fill in the gap for the lack mental health professionals, but rather support them in the overburdening task of catering to a mass of vulnerable populations. Governments should invest in increasing access to traditional mental health services and support alongside ethical frameworks for AI mental health chatbots to ease some of their loads. With proper oversight, collaboration with users and mental health professionals, and ethical frameworks to safeguard user data and privacy, mental health AI chatbots could be a great asset to assisting, rather than replacing, therapists.

## Data Availability

The original contributions presented in the study are included in the article/supplementary material, further inquiries can be directed to the corresponding author.
